# Psychosocial Peer Support to Address Mental Health and Burnout of Health Care Workers Affected by COVID-19: A Qualitative Evaluation

**DOI:** 10.3390/ijerph20054536

**Published:** 2023-03-03

**Authors:** Lea Simms, Katherine E. Ottman, James L. Griffith, Michael G. Knight, Lorenzo Norris, Viktoriya Karakcheyeva, Brandon A. Kohrt

**Affiliations:** 1 Center for Global Mental Health Equity, Department of Psychiatry and Behavioral Sciences, George Washington University, Washington, DC 20037, USA; 2 Medical Faculty Associates, George Washington University, Washington, DC 20037, USA; 3 GW Resiliency & Well-Being Center, School of Medicine and Health Sciences, George Washington University, Washington, DC 20037, USA

**Keywords:** peer support, health care workers, burnout, mental health, COVID-19, qualitative evaluation, health care system, staff wellness

## Abstract

Health care workers in the U.S. are experiencing alarming rates of burnout. Furthermore, the COVID-19 pandemic has worsened this issue. Psychosocial peer-support programs that address general distress and are tailored to health care systems are needed. A Care for Caregivers (CFC) Program was developed at an American metropolitan university hospital and outpatient health care system. The CFC program trains “Peer Caregivers” and managers and has four components: the identification of colleagues in need of support; psychological first aid; linkage to resources; and the promotion of hope among colleagues experiencing demoralization. Qualitative interviews (*n* = 18) were conducted with Peer Caregivers and Managers participating in the initial piloting of the program. Results suggest that the CFC program shifts the organizational culture, teaches staff skills for recognizing and supporting others in distress, and supports those staff who are already providing these services informally. Findings suggest that staff distress resulted primarily from external factors and secondarily from internal organizational stressors. External stressors were exacerbated by the COVID-19 pandemic. Although the program has promise for addressing staff burnout, other organizational efforts are needed to simultaneously promote staff wellness. Ultimately, psychosocial peer support programs for health care workers are feasible and potentially impactful, but also require other systemic changes within a health care system to improve and sustain staff well-being.

## 1. Introduction

### 1.1. Background

Health care workers in the U.S. are experiencing alarming rates of burnout, with 35–54% of nurses and physicians experiencing substantial symptoms of burnout [1]. Burnout is included in the 11th Revision of the International Classification of Diseases (ICD-11) as an occupational phenomenon and is characterized by feelings of energy depletion or exhaustion, increased mental distance from one’s job, or feelings of negativism or cynicism related to one’s job; and reduced professional efficacy [2]. Burnout can result in personal problems for the providers such as poor physical health, relationship problems, and increased mental health issues [3]. Furthermore, burnout can have negative consequences for the health system and for patients. Burnout has been associated with poor organizational functioning (tardiness, absences, increased turnover, etc.) as well as issues with quality and safety such as increased medical errors, poorer patient perceptions of safety, and increased patient mortality [4].

### 1.2. Current Literature

The 2019 National Academy of Medicine consensus report found that burnout is caused by an imbalance in the clinician’s job demands and the available supportive resources in the organization. Work demands that contribute to burnout include excessive workload, unmanageable work schedules, and inadequate staffing; administrative burden; workflow, interruptions, and distractions; inadequate technology usability; time pressure and encroachment on personal time, moral distress, compassion fatigue, and patient factors. In addition to work demands, individual factors such as work–life integration and the combination of personal and professional responsibilities and activities also contribute to burnout. Furthermore, the external environment such as structural changes in the health care industry; the laws, regulations, and standards for the oversight of clinicians; and changing societal values and the clinician–patient relationship, has an impact on health care worker burnout [1].

The COVID-19 pandemic has exacerbated the already existing burnout that many health care workers were experiencing. Research already shows that workers in health care settings have higher prevalence of trauma-related stress symptoms and increasing rates of stress, burnout, anxiety, depression, and insomnia due to the COVID-19 pandemic [5,6,7]. The factors related to the pandemic that have been shown to contribute to distress among health care workers include concern about the spread of the virus, their own health, the health of their loved ones, and changes in the work environment [5]. Furthermore, commingling symptoms of burnout can mask symptoms of more chronic depression and anxiety among health care workers and can be challenging to detect [8,9]. Without intervention, the COVID-19 pandemic will have lasting effects on these caregivers’ mental health and well-being. Therefore, it is imperative that health care organizations address staff well-being.

Psychosocial peer support has emerged as a promising way to mitigate the negative effects of health care worker burnout and trauma and help address compassion fatigue. Several programs have been developed that focus on second victimization and moral injury, which utilize peer-support including the Johns Hopkins University Resilience in Stressful Events (RISE) program. This program recruits and trains peer responders to work with other providers experiencing an adverse clinical event [10]. Although no comprehensive evaluation of the program has been conducted, preliminary findings have shown that RISE is perceived as beneficial to leadership and frontline staff and has contributed to improvements in culture, productivity, and turnover [10]. Similar programs focused on second victimization were implemented in the Brigham and Women’s Hospital, the Los Angeles County-USC Medical Center, and the New York Health and Hospitals system [11]. Limited evaluations have been conducted on these programs. Similarly, a Peer Support Network pilot program was implemented in a midwestern hospital and found that training a team of knowledgeable peer responders contributed to improved compassion fatigue in just six weeks [12]. Although anecdotally successful, these programs focus specifically on supporting health care workers immediately after a stressful clinical event, and there is a need for broader, wellness-based training to prepare staff members to support each other during more general stress. This general stress can be related to the health care worker’s personal lives, organizational and job-related stressors, in addition to adverse clinical events. Furthermore, due to the complex nature of health care worker burnout as well as the localized effects of COVID-19 [5], it is important that peer support programs are tailored to the work-environment and the factors that are causing distress among their specific employees [11]. Given the complexity of health care worker burnout and overlap with symptoms of depression and anxiety, programs additionally need to focus on key strategies for support providers to determine whether colleagues need to be linked with further care from specialized psychological professionals or other organizational resources. There are only limited evaluations of psychosocial peer support programs for health care workers and even fewer qualitative evaluations of these programs. Qualitative research is needed to explain how these types of peer-support programs function within the health system, the causes and contributors to distress, and the effects of COVID-19 on the health care workers in a specific health system.

### 1.3. Context

**Figure 1:**
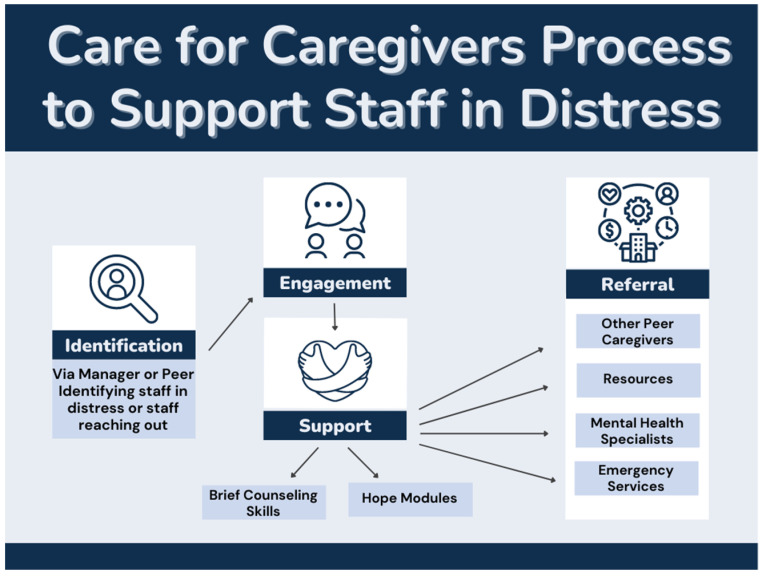
Care for Caregivers process to support staff in distress.

Based on the literature, staff satisfaction surveys, and concerns raised during the COVID-19 health care response, a peer support program—a Care for Caregivers (CFC) service—was developed and implemented at an American metropolitan university hospital and outpatient health care system. This CFC program trains health care managers in supervisory positions and frontline health care staff. The health care staff are given the title of “Peer Caregivers” after the training. CFC trains health care workers and managers to identify colleagues and other staff in need of support, provide brief counseling, and link them to additional Employee Assistance Programs (EAPs) and other resources. Figure 1 depicts the processes of the CFC program for supporting staff in distress. The first component is to identify co-workers who need support. This is done through brainstorming exercises about the signs when colleagues are experiencing distress. The second component is engagement (i.e., how to start conversations in a supportive and nonthreatening manner with colleagues experiencing distress). The third component is the support provided to the co-worker using techniques such as the Hope Module. The fourth module is referrals when needed.

The goal of this study was to evaluate the context, design, and implementation of the CFC Program utilizing qualitative interview data. Additionally, this paper will describe the CFC program in detail so that this program can be replicated in other health care organizations and settings. The research questions this study aims to address are: (1) What is the existing need for staff well-being support and resources at the specific university health care system where CFC was implemented? (2) Does the CFC program address this need? and (3) Is the CFC program the best strategy to address this need?

## 2. Materials and Methods

### 2.1. Study Design

The CFC program was tailored to the specific needs of the university health care system and the curriculum focuses on providing staff with support when they are experiencing any distress, rather than just providing peer support after a patient-related adverse outcome event. The study followed a formative and process evaluation design, incorporating direct observations via interviews and surveys to gather data on the development and implementation of the pilot program [13,14].

The research team reviewed prior staff satisfaction surveys and concerns that were raised by staff before developing the CFC curriculum. Initial stakeholder interviews were conducted to inform the need for the program, potential barriers, curriculum content, and strategies for staff engagement. An initial pilot test of the curriculum was conducted, and the training was improved based on interview feedback from the participants. Once the program was rolled out, feedback from the participants was utilized to continuously improve the content, delivery, and marketing of the program.

### 2.2. Care for Caregivers Training

The CFC program consists of two types of training: the Manager Orientation and the Peer Caregiver Training. The Manager Orientation is a brief 2-h training that introduces managerial level staff, primarily Nurse Managers, to the Peer Caregivers program and briefly outlines how to identify and refer staff in distress. The Peer Caregiver training is a 3 h in-depth training that focuses on the identification of colleagues in need of support; the World Health Organization’s Psychological First Aid curriculum [15]; linkage to EAPs and other resources; and the promotion of hope among colleagues experiencing demoralization using a brief single session intervention entitled the Hope Module [16]. Table 1 provides an overview of the training components. These training components were based on the strong evidence supporting Psychological First Aid [17] as well as key stakeholders within the organization recommending the Hope Modules. Research suggests that scenario-based interactions and post-training supervision is essential to the effectiveness of the training on brief counseling skills [17], which is in line with the stakeholder feedback suggesting that both be included in the curriculum. The Peer Caregiver training curriculum integrated multiple role-playing and group discussion activities. Additionally, ongoing supervision sessions, open to both Peer Caregivers and Managers, began after the conclusion of all training and were conducted by a staff resilience and well-being program within the university’s medical school.

One Managers Orientation and five Peer Caregivers training courses were conducted over a six-month period. Due to the COVID-19 pandemic, only one Peer Caregiver training course was held in-person, while all other courses were delivered remotely using video teleconferencing software. A total of 49 health care staff were trained: 14 staff participated in the Managers Orientation, eight of those managers also participated in the Peer Caregiver training, and 27 staff only participated in the Peer Caregiver training. Of the 49 staff, only three worked in the medical school, and all other participants were part of a faculty medical practice. The training courses were marketed widely through staff newsletters, the organization’s Intranet, dedicated email blasts, presentations at staff town halls, and presentations at the Patient Safety Council and Nurse Managers meetings. Training participants represented a wide range of departments and health care worker roles.

### 2.3. Data Collection and Analysis

Semi-structured qualitative interviews were conducted pre-and post-training. Participants were recruited using nonprobability volunteer sampling where all program participants were invited to be interviewed, and only those who responded were interviewed. Additionally, stakeholders were purposively recruited based on their roles in the university health care system. Participants were given consent forms prior to each interview and were asked for their verbal consent at the beginning of the interviews.

The interview content focused on the participants’ reflections on the environment at the university health care system, the existing and needed well-being resources, and their experience with the CFC training. Interviews were conducted in English and lasted approximately 30 min and were conducted via video teleconferencing software. Audio recordings of the interviews were stored in an encrypted folder. Interviews were first transcribed by the teleconferencing software, transcripts were de-identified, and then the transcripts were edited by two research assistants for accuracy by comparing the audio transcripts. All participants were given unique identifiers that were stored in a password-protected file.

Data analysis followed a thematic approach and was completed using Dedoose 9.0.46 [18]. A codebook was developed with each code having its own unique definition and inclusion/exclusion criteria [19]. The codebook was first developed deductively with one member of the data analysis team (LS) creating pre-established codes based on the research questions. Additional codes were created inductively based on emergent themes within the transcripts and the final codebook can be found in Table 2. Two members of the data analysis team (LS and KO) coded the interviews: they independently coded the transcripts, discussed the reasons for coding disagreements, and adjusted the code definitions and coding strategies to improve agreement. Before starting the final coding, these raters completed an inter-rater reliability (IRR) process. IRR among the two coders reached 0.77 before formal coding was initiated. In total, the data analysis team went through sixty percent of the total transcripts to develop codes until meaning saturation and agreement amongst coders was reached [20]. Final coding was completed on all interviews. The first author (LS) developed summaries of the codes relevant to this manuscript and conducted structured comparisons to identify differences in the perceptions and experiences by the type of participant (Manager, Peer, or Stakeholder) and type of interview (pre- or post-training). To ensure confidentiality, participants were identified using only their identification code, type of participant, and general role within the health care system (Administrator or Clinician).

## 3. Results

### 3.1. Qualitative Findings

Twenty-three qualitative interviews were conducted with 18 participants. Participants included four leaders/administrators in the health care system, four CFC-trained clinical managers, and 10 CFC-trained Peer Caregivers. Five participants were interviewed twice (pre- and post-training) and 13 others such as the stakeholders and those who had limited availability were only interviewed once. All 49 staff who participated in the training were invited to be interviewed, and 35 staff declined to be interviewed by not responding to the invitation. Twelve of the participants were clinicians (e.g., physicians, nurses, medical assistant) and six were administrative staff (e.g., director, coder, administrative assistant). See the full participant profile in Table 3.

#### 3.1.1. Existing Need for Staff Well-Being Support Prior to the CFC Program

**Figure 2:**
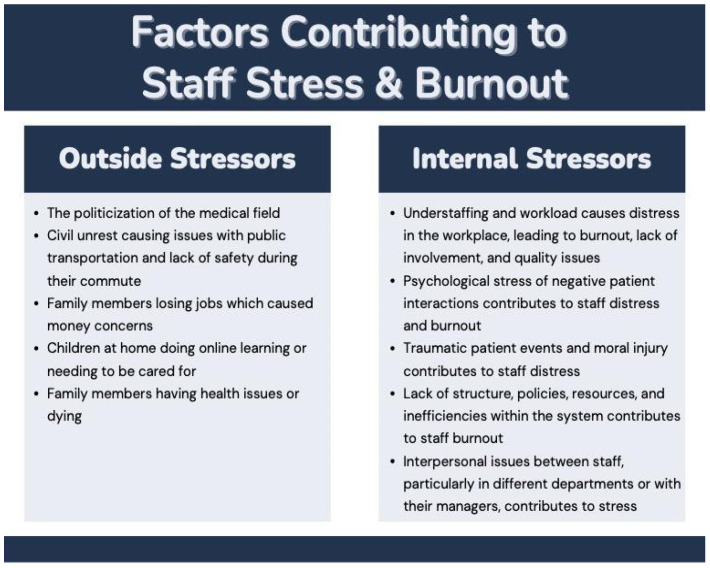
Internal and external factors contributing to staff stress and burnout.

Factors that contribute to staff distress and burnout are a combination of challenges experienced by staff external to the organization (e.g., financial concerns, family demands) as well as internal organizational challenges (e.g., workload, inefficiencies, interpersonal issues), however, most participants expressed external factors as a stronger contributor to staff distress than internal organizational factors. Additionally, the findings suggest that outside stressors were exacerbated by the COVID-19 pandemic. Figure 2 presents a summary of the key internal and external stressors and Table 4 contains the narrative quotes.

Findings suggest that prior to the CFC program, there was already an existing informal referral network for staff in distress. Participants discussed how they themselves, other peer staff members, or their managers already acted as an informal support network for staff in distress. Formalizing and providing support to those informal “Peer Caregivers” and those in more formalized management positions is essential to ensure quality, demonstrate organizational support, disperse the responsibility to more “Peer Caregivers”, and mitigate burnout among those staff who are already supporting their fellow staff.

*“So, I feel like with the nurses, we’ve got a couple good nurses who are really good listeners. But that’s also not always good for them, because sometimes too much is put on someone and then they might be the one that’s coming to me saying there’s a lot going on, you know, but yeah, the nurses, the nurses are pretty tight”.*

(M05, Clinician, Manager)

#### 3.1.2. Existing Staff Well-Being Resources and Services Prior to the CFC Program

The university health care system’s previous initiatives, trainings, and existing resources to address staff well-being and staff’s experiences were explored. Most of the participants interviewed had not received prior training on mental health and wellness while employed at the university health care system. The few participants that did recall wellness training described them as intending to boost staff morale and encourage better communication among staff. Some participants described these prior wellness training sessions as “disingenuous”, that they did not dig deep enough into the staff needs and that one training was not enough, and that wellness needs to be integrated into the health care system culture to make a difference.

*“I thought [the culture training] was okay, [but] somewhat disingenuous. Many people come at this with sort of a jaded attitude, so I think you know more than boostering, you have to kind of dig a little deeper…you have to find out what are people’s problems with the [university health care system]”.*

(P02, Clinician, Peer Caregiver)

As for organization-wide wellness initiatives other than training, only participants (primarily the four stakeholders) who were involved as leaders in developing staff wellness initiatives were aware of them and the other participants interviewed did not mention any wellness initiatives that they were aware of. Of the participants who did mention their involvement in wellness initiatives, they listed initiatives such as a wellness committee, a virtual covid peer-support group, a speakers’ bureau where they discussed wellness-related topics, student and resident wellness initiatives, staff survey on wellness needs, a creation of a list of community wellness resources, a workplace violence initiative, and the newly formed medical school resiliency and well-being program. This signifies that there is a need for organization-wide staff wellness initiatives to be more widely marketed to raise awareness of these opportunities and continue to demonstrate the shift toward a culture of wellness within the university health care system.

*“Interviewer: Okay. So, have you been involved in any staff wellbeing initiatives at [the university health care system]? And if so, can you describe them?*

*Participant: Um, I’m gonna say, no, um not anything that I can recall. Not anything that I can recall”.*

(P10, Administrative, Peer Caregiver)

A few participants mentioned being involved in departmental wellness initiatives such as departmental staff days, 2-min of mindfulness as a department at the beginning of the pandemic, a team yoga class, adding color to team spaces, and more general emphasis of teamwork, support, and wellness among their departments.

*“So, for my team, one of the things that we did was a yoga class, so we did it as a team and I thought that that was really great to do because we got to know each other in a different way, it was something that kind of helped with team morale, it was a way of also keeping us accountable for taking that break or staying mentally and physically healthy”.*

(P07, Administrative, Peer Caregiver)

The university health care system has an Employee Assistance Program (EAP) where all employees have access to a wide variety of resources. In general, Managers tended to be more knowledgeable than Peers and Stakeholders about EAP resources, but they had mixed experiences utilizing the services themselves or referring staff.

*“I know we do have a resource of EAP. What they offer and what they do … I’m not sure it meets the needs of our current employee population”.*

(M02, Clinician, Manager)

*“And that was one of the good things that I saw that the EAP has, there’s a lot of resources there for finances for counseling, or anything like that … And I didn’t want to pry but I just wanted to make, you know, just make sure [my staff member in distress was] aware that, hey, try this resource. They’re there to help you”*

(M01, Clinician, Manager)

Non-management staff knew that EAP benefits and resources existed, however, beyond that, they were not knowledgeable about what was provided. In general, all participants knew that there was a phone number or website where they could find the employee resources. Staff learned about the EAP resources during orientation, from their managers, all staff emails, or from information sent out during the open benefit enrollment period.

*“Um, um, I’m gonna be honest, if they’ve mentioned [the EAP resources] before, if somebody really asked me, if we had it, I probably wouldn’t have denied it. But I wouldn’t have been able to confidently confirm it either”.*

(P10, Administrative, Peer Caregiver)

Among those who did know something about the EAP benefits, by far the most common EAP benefit the participants were aware of was the free counseling sessions. A few participants said that there was some hesitancy around using and referring staff to the EAP benefits, particularly counseling, because of the fear of retaliation from their employer, stigma, and information “staying in your file”. Other specific resources that some participants mentioned being aware of included free access to the online and app-based meditation site HeadSpace and other wellness apps; legal support; funeral resources; child/elder care services; free exercise classes; and the online nutrition shop. Participants also mentioned outside resources they referred their colleagues to such as group therapy, hobbies, local events, legal services, and pastoral care.

*“I think that’s a real concern that here’s like I don’t want to go to HR and somehow, it’s in my file. Or I don’t want to go anywhere close, and someone sees me, so just a fear and the stigma about getting help is a big one, so I think sometimes just offering them inside help, but then also like okay, and if you don’t feel comfortable, here are some options too”.*

(P07, Administrative, Peer Caregiver)

In addition to the free counseling via the EAP, some participants mentioned that financial concerns, specifically the lack of employee insurance coverage, was a barrier to seeking outside mental health counseling when more intensive or long-term care is needed from a psychologist or psychiatrist.

*“To be honest, I don’t even know if our health insurance covers therapy, largely because I haven’t looked into it. And also, because it’s terrible. So, I assume it doesn’t”.*

(P11, Administrative, Peer Caregiver)

#### 3.1.3. Benefits of the CFC Program and Strengths of the Training

Some common themes arose about how the CFC program will benefit the university health care system. The CFC program will shift the organizational culture by signifying the leadership’s commitment and creating a more supportive environment for staff, resulting in better job performance and patient care.

*“We want to be available to people, but even just knowing that a program like this exists, I feel like will help people to feel supported so that even if they don’t need it right now, or ever, possibly, just knowing that [the university health care system] cares to the extent that they’ve developed this program and their fellow colleagues are participating in it, I feel like that itself goes a long way”.*

(P03, Clinician, Peer Caregiver)

Participants discussed how the staff that supported others were burnt out, so it will be helpful to have a wider net of Peer Caregivers and someone to support them. Furthermore, having Peer Caregivers in different departments will help to address the privacy and confidentiality concerns some staff had with talking to staff in their departments or their own managers.

*“Sometimes I wish I had somebody else here that I could go to, that will want to extend to me what I love extending up, do you know what I mean? So, I can find my [participants name], if I see their badge, you know, if I know that we have this program out here, I don’t always have to be the one helping. I can actually go to someone for you know, just support”.*

(P10, Administrative, Peer Caregiver)

A few participants discussed how the training re-ignited their drive to support colleagues in distress and saw it as part of their role.

*“[the CFC training] came at a perfect time for me because I was feeling really burnt out. And, it kind of just like, rejuvenated that feeling of like, this is like a service, this is what you need to do. This is kind of how your role plays itself out. So, I think it was like a great reminder of that, for me, especially when we’re on like round 12 of the pandemic or something”.*

(P11, Administrative, Peer Caregiver)

Themes also arose related to the most helpful skills and knowledge that the participants gained from the Peer Caregiver training and Managers Orientation. Specifically, the participants mentioned that the training would help them to better identify their colleagues in distress, improved their listening skills, improved their ability to communicate, gave them tips about the dos and do nots of providing peer counseling, and made them feel more confident in having difficult conversations. A few participants discussed how the training helped them recognize that it was acceptable to create personal boundaries.

*“I think just thinking about how it shows up in the workplace and how they want support is important. Um, because everyone doesn’t want the same type of support and recognizing that and being able to pivot when necessary”.*

(P07, Administrative, Peer Caregiver)

Another theme was that the training helped to make those already providing support to colleagues in distress feel less overwhelmed and prepared for these conversations. Formalizing and clarifying the role of Peer Caregivers helped to make the supporting staff feel less overwhelmed. Additionally, raising awareness about referral resources helped to alleviate the stress of the Managers and Peer Caregivers because they had something to provide staff as a next step.

*“But to me that was the most enlightening part to know that, after you’ve done your part, you can always give further instructions for further help”.*

(M01, Clinician, Manager)

In general, the participants also gave positive feedback on the training logistics. Most commonly, the participants mentioned that the interactive activities that were built into the training helped to improve their skills and made the training engaging. A few participants said that having a diverse group of staff attend the training from different levels and professions within the organization helped to make the training feel more inclusive and made the discussions interesting.

*“I think [the role plays] really kind of helped work through, you know, like real life scenarios and how to implement the training, in a way that, you know, just listening to a presentation wouldn’t work for me”.*

(P05, Administrative, Peer Caregiver)

#### 3.1.4. Challenges and Potential Barriers for the CFC Program

The most frequently cited potential concern that participants had with the CFC program was that they anticipated some general skepticism from staff about the CFC program. Some of the reasons for this skepticism included: (1) resistance to the program because of it merely being a new program; (2) staff might not appreciate the program until they need it so might not immediately see the value in the program; (3) because staff work within the health care field, they might be more critical of services; and (4) staff might be aware of other failed initiatives.

*“I think they’re gonna think it’s good. But a lot of people are gonna say, but I don’t need it. But it’s not for me. And, but I do believe that is going to be that background thing in your head, like, I do have this… It’s always good to know that you have something, but I’m not sure what it’s going to take to make it real for people to share, if that makes sense”.*

(P10, Administrative, Peer Caregiver)

Another potential barrier discussed was the staff’s fear that the information disclosed to Peer Caregivers might not remain confidential and the fear of consequences (such as being fired) for the information they shared with the Peer Caregivers. Because this was brought up in the stakeholder interviews, it was incorporated into the Managers Orientation and Peer Caregiver training curriculum. However, even with messaging around respecting confidentiality and privacy in the training, the participants continued to have concerns that it would impact the staff’s decision to speak with a Peer Caregiver or their Manager.

*“I think there may be some initial maybe concern or hesitancy but if there’s like hopefully there’s some word of mouth, or you know if there’s reassurance on confidentiality, I think the main concern is like privacy … I think it would depend on how its presented and maybe like word of mouth … And it may be initially challenging to get people to use something like that, within the workplace, yeah”.*

(K04, Clinician, Stakeholder)

Another theme was that some participants anticipated the Peer Caregiver’s skills, or lack thereof, as potential barriers to the program. A few participants discussed how people could not be trained for every situation, the retention of content might be a challenge, and complex situations might be outside the Peer Caregiver’s scope. Others talked about the importance of carefully recruiting Peer Caregivers and avoiding training those without the innate skills and personality to support others.

*“Um, I mean, I think that maybe there are some people who would not be as appropriate to be trained in these types of things just because of either, you know, their personalities or their workload may not permit for those types of interactions. But otherwise, I think it’s good for most people”.*

(P05, Administrative, Peer Caregiver)

An anticipated barrier to program implementation is that staff will not have the time to become Peer Caregivers, support staff as Peer Caregivers, or utilize the Peer Caregiver services when they are in distress:

*“I’m, afraid that we may have that opportunity for them to actually go and talk, but they won’t have the time to be able to do that. So that, that would be one of my fears, and, might be one reason why is, they may not be encouraged to actually be a part of it, because their time is already consumed”.*

(M01, Clinician, Manager)

Two participants mentioned that one barrier to engagement in the program was the organizational culture within MFA, describing it as “sour” and “a lack of social trust”.

*“I think that because the culture is a little bit sour, that, you know, people are not often trying to go out of their way to be helpful, just because they already feel so taxed with everything else that they have to do”.*

(P05, Administrative, Peer Caregiver)

Another challenge for the implementation of the CFC program mentioned by a few participants is the lack of physical space in the clinical office building for private conversations. Where to have private conversations was addressed during the training because it came up during the stakeholder interviews, however, it was mentioned by a few participants in later interviews, even with the content incorporated into the training.

*“I worry a little bit about where to like talk to people. I think yeah, I mean I know space is like super short in the hospital everywhere for everybody, so yeah, I think that’s a big one of like, how do you have private conversations?”*

(K03, Clinician, Stakeholder)

#### 3.1.5. Suggestions for Improvement to the CFC Program

Participants were asked about how the CFC program could be improved, and the most common response was that raising staff awareness is essential to the success of the CFC program. Marketing the program encourages staff to be trained and it also encourages staff to utilize the Peer Caregivers as a resource. Ideas for raising awareness included recognizing Peer Caregivers in the staff newsletter, sending out designated emails about the program, encouraging those who have been trained to make announcements during team/department meetings, social media campaigns, posters in staff breaks, announcements in staff town halls, including the training in staff orientation, and having a “Peer Caregiver” sticker on staff badges who had been trained. Many of these suggestions were incorporated into the CFC program marketing as they were suggested.

*“I think it’s more so exposure, the more people know about it, is, the more they will want to be involved with it, those who want to be from the side of the caregiver, to those who want care, to those who need care, when they realize that there is a resource available, I think that it’s going to open up, you know, the floodgates, in essence, for people to come in”.*

(P09, Administrative, Peer Caregiver)

Some participants gave feedback on the format and logistics of the CFC training. Participants suggested the training group size should range from 4–15 people. A few participants had suggestions related to the training length and structure. Suggestions included expanding the training to three hours (rather than 2) to allow for more role-play and an “open forum” for participants to ask questions as well as breaking up the training into shorter sessions spread over three days.

*“I think the [training] could actually be maybe a little bit longer and have, you know, more like hands on type of stuff. Because that was really very good. And I just felt like, even though you know, going into it, that the two hours is, you know, like, oh my gosh, and most meetings are half an hour, an hour, and it’s always gonna be a long time, it actually went by very quickly”.*

(P05, Administrative, Peer Caregiver)

Participants also suggested additional resources that could be incorporated into the training. Suggestions included shortening pre-work videos into shorter clips and “cheat sheets” to be referred to when having a conversation with staff in distress. The “cheat sheets” could include common scenarios and statements from staff in distress and a checklist of signs of distress along with possible responses and next steps.

*“My suggestion is to have sort of a cheat sheet, so if we are going to sit down with somebody, I like to be prepared, so I’d want to probably memorize that or at least be really familiar, so that we can effectively help. I think, relying on your sort of personality and skills to you know help others isn’t always the greatest of ideas, you want to have a structure”.*

(P02, Clinician, Peer Caregiver)

Another theme was the importance of training. Ideas for new audiences that should be trained as Peer Caregivers included medical students, residents, and making it available to all staff in both management and not in management roles.

*“Probably if others have access to the training as well. Not just those on a managerial level, I think it will be helpful for others”.*

(M04, Clinician, Manager)

Participants discussed the need to carefully assign staff to Peer Caregivers to address hierarchical and confidentiality concerns. Suggestions were to have Peer Caregivers available in other departments or to have multiple Peer Caregivers in each department, assigning one Peer Caregiver to a group of staff, a unit, or a department, or to have group peer counseling sessions, available to anyone in the Peer Caregiver’s department, in addition to the option to have one-on-one conversations.

*“Because of the privacy concerns and confidentiality like if people are not paired with people within their department … I think that may be better and people you don’t see like every day because I think that was a concern … having space between whoever you are going to talk”.*

(K04, Clinician, Stakeholder)

A few participants highlighted the importance of ongoing support and training, after the initial Peer Caregiver and Manager training courses. Participants suggested that these could be either formal monthly or bimonthly booster sessions with additional training and collaboration among the Peer Caregivers to share experiences or informal mentorship with a structure at the programmatic level for that mentorship to take place. In response to this feedback, regular supervision sessions were built into the program.

*“I think monthly or bimonthly booster calls, where maybe a particular topic is discussed … it might just be an opportunity for some like ongoing training collaboration if, perhaps, you know, there’s a way to share … in a confidential way about experiences people have had as part of the program, as the Peer Caregiver. Things like that might be helpful”.*

(P03, Clinician, Peer Caregiver)

#### 3.1.6. Suggestions for Organizational Changes, Resources & Services

Participants mentioned organizational changes, resources and services, outside of the CFC program that could improve staff distress. Three common themes arose. First, the participants discussed that there was a need to create a culture of recognition and valuing staff within the organization. Concrete suggestions included sending cards for important events (birthdays, work anniversaries, condolence cards for bereavement), monthly kudo or recognition emails, and following up on feedback.

*“Oh, I believe we need to start pouring into our employees, which, in my understanding, we have not done in many years at the MFA … And I don’t mean pour into them financially, right? I mean rewards and recognition, creating a culture of—a just culture, a culture where they can speak up and they feel valued and that they’re heard”.*

(M02, Clinician, Manager)

Second, there is a need for more intensive referral resources to refer staff who are experiencing more complex issues than the Peer Caregivers and Managers are equipped to handle. Participants suggested social workers, an occupational health department, emergency services, and/or psychiatrists.

*“It would be nice to have perhaps, this is like a dream world, employee based like social workers or a health department. Maybe our occupational department has some kind of branch to it”.*

(M05, Clinician, Manager)

The last common theme that came up regarding the organizational changes and resources was that there was a need for more communication to staff about what wellness resources were available. Specifically, participants discussed reviewing wellness policies with staff on a regular basis, reminders about who staff can talk to when they are in distress, and general reminders about the available resources and for staff to prioritize their wellness.

*“But I also think that in terms of, of wellness overall, that that’s something that is lacking is kind of a review of policies and things from a higher level that may benefit employees, if there were some changes to policy”.*

(P05, Administrative, Peer Caregiver)

**Figure 3:**
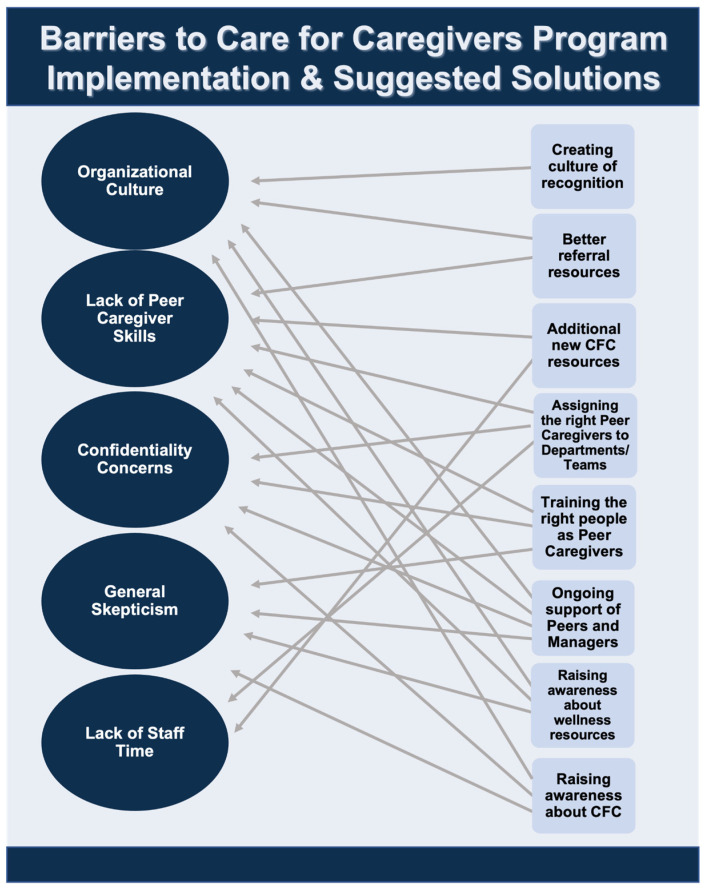
Barriers to the Care for Caregivers program implementation and suggested solutions from participants.

Figure 3 shows a diagram depicting the major themes outlined by participants of the potential barriers to the implementation of the CFC program as well as the suggested solutions.

Other organizational changes to improve staff wellness that were suggested by only one participant included more flexibility on task deadlines, scheduling, and tele-work, a relaxation room for staff, mandatory lunch breaks, giving staff a “wellness day-off”, ensuring work was more evenly dispersed, more timely response to staff requests (like changes to schedules, requests for help, and vacation time), manager training on management skills, and a general culture shift where working after hours was discouraged.

## 4. Discussion

The qualitative interview findings suggest that there is a strong need for the CFC program at this metropolitan university health care system. Contrary to what the researchers expected to find, external factors outside of the organization such as the participants work–life integration and external environmental factors were more commonly discussed as contributing to staff distress than internal organizational factors. This differs from previous research that mostly emphasizes internal organizational issues as the main cause of burnout [1]. Furthermore, this study was conducted during the midst of the COVID-19 pandemic, and the findings suggest that although the external stressors already existed, the pandemic exacerbated these stressors for the staff. Because of the economic and public health devastation left in the wake of the pandemic [21], this is to be expected, and likely explains why external stressors were emphasized by the participants and internal organizational stressors were highlighted secondarily. Internal organizational issues such as the lack of structure and policies, understaffing, staff workload, feelings of underappreciation, interpersonal issues among coworkers and patients, and moral injury were discussed as contributors to staff distress.

Overall, the participants had a positive experience with the Peer Caregiver training and Managers Orientation. Specifically, the participants appreciated the interactive elements. The findings suggest that the training will help participants better recognize distress, have difficult conversations, listen actively, set personal boundaries, and provide referral resources. Although participants generally thought it would be well-received by other staff, general skepticism, confidentiality concerns, the Peer Caregiver’s skills, staff having competing priorities, staff’s large workload, and lack of physical space for private conversations were anticipated to be barriers to the CFC program adoption. Marketing and raising awareness of the CFC program was found to be essential to the program’s success and adoption within the organization.

General skepticism to new programs was discussed by a number of participants and resistance to change within organizations and its relationship with burnout is a highly researched topic. A 2017 study of Canadian health care staff who were involved in a large-scale organizational change found that job control/autonomy and supervisor support protected from the factors leading to burnout caused by organizational change [22]. This supports the finding that the CFC training of managers can help to mitigate burnout caused by pandemic-related organizational change as well as the change from implementing the CFC program and general resistance to this new program. Furthermore, increasing the staff’s job control through addressing some of the internal organizational factors contributing to stress and burnout as previously discussed will help to reduce exhaustion and cynicism, and increase professional efficacy.

Peer Caregivers, Managers, and Stakeholders described one of the biggest benefits of the CFC program as shifting the organizational culture of the university health care system by signifying the leadership’s commitment to staff wellness and creating a more supportive environment for staff. The results of this shift in organizational culture will result in better job performance and ultimately, better patient care. This is similar to findings from other peer-to-peer health care worker support programs where they had a positive effect on organizational culture [8,9].

An interesting finding was that an informal support network of staff providing support to their colleagues in distress already existed prior to the CFC program. This is in-line with the Agency for Health Care Research and Quality’s “Care for the Caregiver Program Implementation Guide” where they suggest identifying “natural” second-victim supporters and those individuals who are already supporting their staff [23]. Our qualitative findings suggest that formalizing this network and providing those staff with training and resources will help to disperse the responsibility and make staff more effective at providing peer support, thus allowing the support of other staff to feel less overwhelming.

**Figure 4:**
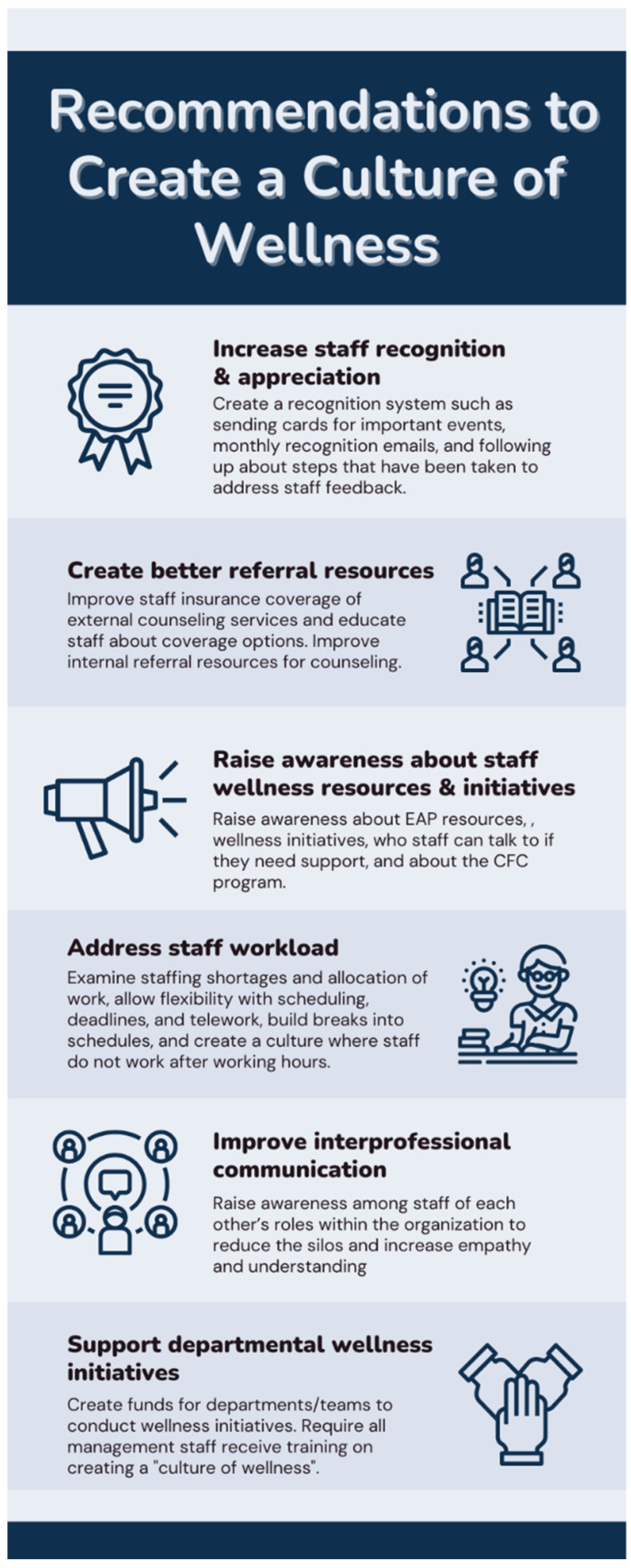
Recommendations to create an institutional culture of wellness.

For the CFC program to be successful, there is a need for more widespread organizational efforts from the university health care system to address staff well-being. This “culture of wellness”, described by many participants, can help to address many of the factors that contribute to staff burnout and can work in conjunction with the CFC program. See Figure 4 for a list of recommendations, compiled by the authors, to foster a “culture of wellness” within the organization. These recommendations are a combination of explicitly stated suggestions from the interview participants (see Figure 3) and the recommendations from researchers based on problems identified by the participants. Additionally, by merely having the CFC program, participants felt that the organization valued them. This, as well as the other findings about the benefits of this program to the organization, suggest that other health care systems should consider implementing similar psychosocial peer-support programs for their staff.

### 4.1. Limitations

There were several limitations to the qualitative portion of the study. Because of the volunteer sampling method, one limitation of the study is self-selection bias. Those program participants who volunteered to be interviewed might have different views of the CFC program from those who did not volunteer. Similarly, with hand-selecting of the stakeholders, there might be some bias in who was invited to be interviewed. Additionally, there was an over representation of the faculty clinical practice employees in both the training attendance and qualitative interview participants, meaning that the findings might be more relevant to the faculty clinical practice rather than to staff directly employed by the medical school (because those employed by the medical school have greater benefits and employment stability than health care workers in the faculty clinical practice). Another limitation is that some program participants were only available to be interviewed once after the training, so their responses might have been impacted by having already attended the training.

### 4.2. Future Research Directions

Subsequent evaluations of the CFC program should focus on short-term and long-term impacts of the training on staff burnout and patient care. Meta-analysis comparing different peer-support programs and training curricula are needed to find the most effective program elements. Further research is needed to independently study external factors to the workplace causing stress and burnout within the workforce. In general, other institutions and health systems should be encouraged to adopt and customize staff wellness programs, and those with peer support elements are recommended.

## 5. Conclusions

These findings suggest that the Care for Caregivers program implemented at a university health care system helped to shift the organizational culture, taught staff skills for recognizing and supporting distress, and supported the staff who were already providing these services (e.g., Managers and an informal network of staff who support others). Furthermore, the Care for Caregivers program was found to be needed to address the staff distress resulting primarily from external factors as well as internal organizational stressors that were exacerbated by the COVID-19 pandemic. Although the program has promise for addressing staff burnout, other organizational efforts are needed to simultaneously address organizational contributors to staff distress.

## Figures and Tables

**Table 1:** Care for Caregivers training components.

Managers’ Orientation to Care for Caregivers	Overview of Care for Caregivers Program; Orientation to proactive identification and referral of care.
Peer Caregiver Training	Proactive Identification of colleagues in distress; Psychological First Aid (PFA)—Evidence-supported approach to supporting people who have experienced an individual or collective adverse event; Hope Modules—Brief psychological support conversations (20–30 min) when individual is exhibiting demoralization; “Look, Listen, Link” to additional EAP or other resources.
Ongoing Supervision/Booster Sessions	Allow for continued skills development for Peer Caregivers; Create community and peer support for Peer Caregivers.

**Table 2:** Qualitative codes and descriptions.

Code Name	Code Description
Challenges/Needs for Staff Well-being Support	Staff well-being or mental health needs and challenges to supporting staff in distress prior to the CFC program; causes and types of distress observed in the workplace; can include organizational, personal, or larger societal challenges and causes. If discussing resources/services that already exist, code to “Existing Staff Well-being Resources/Services” code, if discussing suggestions for resources/services, code to “suggested Resources/Services”.
Existing Staff Well-being Resources/Services	Utilization or awareness of staff well-being resources/services at MFA or SMHS that existed prior to the CFC program; can include mental health or general staff wellness, can include EAP resources or others; informal methods of supporting other staff; critiques of existing well-being resources; suggestions for needed resources should be coded to “Suggested Resources/Services” code.
Suggested Resources, Services, Organizational Changes	Suggestions for staff well-being resources or services that can be implemented at MFA or SMHS; suggestions for organizational changes that could be made to reduce distress. This code does not include anything about the CFC training, just about suggestions for MFA/SMHS to change.
Strengths and Benefits of CFC	Includes positive descriptive language of the training and training components; what they liked about the training or were the strengths of the training; benefits of the CFC training to the staff member, MFA/SMHS, patients, or larger community/public; expected behavior change after completing the training; examples include “I liked the interactivity”, “this training will help me be a better manager”, “this training makes MFA a better place to work”, “this training prevents issues before they go to HR”.
Challenges/Barriers/Gaps of CFC	Challenges/barriers for CFC adoption or the receptivity of staff; gaps or issues within the CFC training related to content, implementation, etc.; if discussing suggestions for improvement to CFC training, code to “Suggested Modifications and Improvements to CFC”, if discussing gaps outside of CFC scope, code to “Challenges/Needs for Staff Well-being Support”
Suggested Modifications and Improvements to CFC	Suggested changes or improvements that can be made to the CFC content, delivery, reach, etc.; if merely discussing the issues, code to “Challenges/Barriers/Gaps of CFC”, if discussing organizational improvements outside of CFC scope, code to “Suggested Resources, Services, Organizational Changes”

**Table 3:** Participant profile.

Participant	Role (Clinician/ Administrative)	Type (Stakeholder, Manager, Peer Caregiver)	Training Attended (N/A, Manger Training, Peer Training)	Interview (Formative Stakeholder Interview, Pre-Training, or Post-Training)
K01	Clinician	Stakeholder	N/A	Formative stakeholder interview
K02	Clinician	Stakeholder	N/A	Formative stakeholder interview
K03	Clinician	Stakeholder	N/A	Formative stakeholder interview
K04	Clinician	Stakeholder	N/A	Formative stakeholder interview
M01	Clinician	Manager	Manger and peer trainings	Pre- and post-training
M02	Clinician	Manager	Manger and peer trainings	Pre- and post-training
M04	Clinician	Manager	Manger and peer trainings	Pre- and post-training
M05	Clinician	Manager	Manger and peer trainings	Pre- and post-training
P01	Clinician	Peer Caregiver	Peer training	Pre-training
P02	Clinician	Peer Caregiver	Peer training	Pre- and post-training
P03	Clinician	Peer Caregiver	Peer training	Post-training
P05	Administrative	Peer Caregiver	Peer training	Pre- and post-training
P07	Administrative	Peer Caregiver	Peer training	Pre- and post-training
P08	Clinician	Peer Caregiver	Peer training	Pre-training
P09	Administrative	Peer Caregiver	Peer training	Post-training
P10	Administrative	Peer Caregiver	Peer training	Post-training
P11	Administrative	Peer Caregiver	Peer training	Post-training
P12	Administrative	Peer Caregiver	Peer training	Post-training

**Table 4:** Workplace and professional stressors contributing to burnout.

*“And then you’re in this role that is very, very normally is a very prestigious thing to be a doctor, and all of a sudden, everything about your career and what you’ve devoted your life to, is being like politicized on TV, and there’s like, Are you right? Do you know, like, suddenly, it’s just like no place feel safe for you?”* (P11, Clinician, Peer Caregiver)
*“So, it’s just a domino effect when we can’t support them and we’re asking them to do more, and then they walk into an environment that’s not very friendly outside of our walls”.* (M02, Clinician, Manager)
*“Sometimes staff in issues getting along and that is not something that that in terms of getting along that is a big problem. But of course, anytime you’re dealing with humanity or you’re dealing with people, you can always have the possibility of conflict. You know, somebody don’t like something somebody don’t like the way somebody do the things you know, so, yes, we see those type of stuff”.* (P09, Administrative, Peer Caregiver)
*“I think the biggest barrier is that everyone’s burnt out, so I know it’s hard for anybody to get a break, because everyone needs one. So, then you feel bad asking for it, especially if you see your supervisor burnt out and your supervisor’s supervisor burnt out and you’re like I’m burnt out, but how can I say anything because the line is burnt out. I think that’s been the most difficult part”.* (P07, Administrative, Peer Caregiver)
*“No one who walks through the door here is feeling well, really, and, you know, none of the patients who come here or, you know, want to come here, it’s because they’re having some sort of a problem. So, I think that sometimes they can be irritable and confrontational. And I think that, you know, when you’re in that environment, day to day as a staff member who’s interacting with them directly, that it can become toxic, and, you know, kind of exhausting”.* (P05, Administrative, Peer Caregiver)
*“It’s hard you know, you’re watching people die, we’re not in the business to watch people die we are in the business of helping people. It’s hard when you can’t help them and they’re going to die from it”.* (P02, Clinician, Peer Caregiver)
*“Just like the inefficiencies of the workplace like adding up to lead to a lot of um, yeah, a lot of anger”.* (K03, Clinician, Stakeholder)
*“Because it’s not always work either sometime back home, you know, sometimes we carry those personal things, and we say, the home at home, they work at work, but we spend a lot of time to work. And we call it our work family sometimes, right, because we spend a lot of time with those people that we work with. So sometimes it really is hard to cover, you know, draw that line and have that separation”.* (P10, Administrative, Peer Caregiver)

## Data Availability

Data are available from the authors upon reasonable request.
